# Packaging of DNA Integrated with Metal Nanoparticles in Solution

**DOI:** 10.3390/e25071052

**Published:** 2023-07-12

**Authors:** Nina Kasyanenko, Andrei Baryshev, Daria Artamonova, Petr Sokolov

**Affiliations:** Faculty of Physics, Saint Petersburg State University, Saint Petersburg 199034, Russia; andry-barash@mail.ru (A.B.); st077204@student.spbu.ru (D.A.); p.a.sokolov@spbu.ru (P.S.)

**Keywords:** DNA conjugates with metal nanoparticles, silver nanoparticles, gold nanoparticles, platinum nanoparticles, DNA rigidity, DNA condensation

## Abstract

The transformation of high-molecular DNA from a random swollen coil in a solution to a discrete nanosized particle with the ordered packaging of a rigid and highly charged double-stranded molecule is one of the amazing phenomena of polymer physics. DNA condensation is a well-known phenomenon in biological systems, yet its molecular mechanism is not clear. Understanding the processes occurring in vivo is necessary for the usage of DNA in the fabrication of new biologically significant nanostructures. Entropy plays a very important role in DNA condensation. DNA conjugates with metal nanoparticles are useful in various fields of nanotechnology. In particular, they can serve as a basis for creating multicomponent nanoplatforms for theranostics. DNA must be in a compact state in such constructions. In this paper, we tested the methods of DNA integration with silver, gold and palladium nanoparticles and analyzed the properties of DNA conjugates with metal nanoparticles using the methods of atomic force microscopy, spectroscopy, viscometry and dynamic light scattering. DNA size, stability and rigidity (persistence length), as well as plasmon resonance peaks in the absorption spectra of systems were studied. The methods for DNA condensation with metal nanoparticles were analyzed.

## 1. Introduction

Entropy plays an important role in processes, such as the packaging of highly charged and rigid double-stranded DNA in the heads of bacteriophages, chromatin and DNA condensation in vitro. The estimation of the conformational entropic penalty associated with packaging double-stranded DNA into different structures is a subject of controversy. DNA condensation is widely used in modern biotechnologies to create various nanostructures. Understanding the molecular mechanism of this process for DNA associated with other agents is an urgent task.

DNA nanotechnology provides well-controlled templates for self-assembling materials at the nanoscale. Usually, DNA-based technologies like DNA origami, use the predictable base pairing [1,2,3,4]. However, there are also strategies taking advantage of electrostatic or other non-specific interactions in DNA solutions. Such approaches use high-molecular DNA.

By combining metal nanoparticles (NPs) with DNA, it is possible to form new materials with unique electronic and optical properties [5,6,7]. Moreover, after the addition of biologically active substances and fluorophores, such structures allow for the fabrication of complex nanoplatforms for applications in different therapeutic and diagnostic areas [8,9,10]. The unique properties of noble metal NPs, such as localized surface plasmon resonance in the optic area and an enhancement of electromagnetic fields around nanoparticles, are especially relevant. This effect is currently used, for example, in methods based on giant Raman scattering (SERS and TERS) [8,9,10,11]. Noble metal nanoparticles can modify, supplement and make antitumor agents safer and more effective. They can also be used to visualize tumors, to deliver drugs to cancer cells and to enhance the effect of existing medicines [12,13,14,15].

Gold NPs have undeniable advantages in biomedical applications over other types of nanoparticles due to their chemical stability and low toxicity. Nanoparticles have the capacity to enter cells by themselves, but they also carry other molecules through the lipid bilayer. They can be utilized in the cellular delivery of substances like small drugs. Conjugates of high-molecular DNA with metal nanoparticles must be in a compact state for them to penetrate into the cell. In addition, the field enhancement effect reaches its maximum between two closely spaced nanoparticles. This can be achieved by causing DNA condensation after its connection with metal nanoparticles. To ensure DNA compaction in such structures, a well-known phenomenon of DNA condensation in solution can be used. This is the main goal of our research.

Polycations, multivalent ions like polyamines and cobalt hexamine, can cause DNA to undergo a sharp condensation phase transition in solutions [16,17,18]. Toroids can be formed through the careful addition of a condensing agent into solutions with a small concentration of DNA with a low ionic strength. It is especially surprising that the DNA is in the B-form in such condensed structures [18]. DNA condensation is accompanied by a change in the entropy of the system. The molecular mechanism of DNA condensation is still unclear.

The assembly of DNA with gold nanoparticles (AuNPs) was carried out previously in different ways [19,20,21,22,23,24,25,26]. To minimize the chemicals in the preparation of DNA conjugates with NPs, we reduce metal ions via their binding to synthetic polymers with the further combination of polycation–NPs–DNA in nanosized particles. The joint usage of gold and silver nanoparticles in such systems is applied. For comparison, the reduction in metal ions after their binding to DNA was performed using chemicals.

## 2. Materials and Methods

### 2.1. Materials

**Figure 1:**
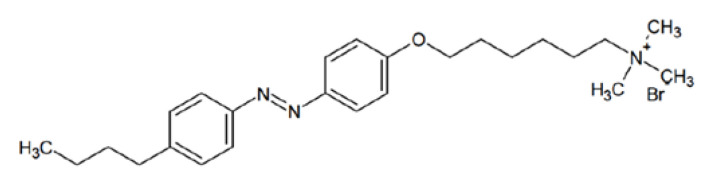
Structure of surfactant Azo-TMAB.

In this work, the salts AgNO_3_, K_2_AuCl_4_ and K_2_PdCl_4_ as sources of metal ions in water solutions and NaBH_4_ as a reducing agent were used. No nanoparticle stabilizers were introduced into the solution when synthesizing nanoparticles by reducing the ions associated with the DNA molecule. A commercial sample of high-molecular calf thymus DNA with a molecular mass of 9 × 10^6^ as determined by the value of intrinsic viscosity of DNA in 0,15 M NaCl was used. The chemicals were purchased from Sigma-Aldrich. The polymer poly [2-(dimethylamino)ethyl methacrylate (poly-DMAEMA), the copolymer of DMAEM and poly [2-deoxy-2-methacrylamido-D-glucose (MAG): poly[MAG–DMAEMA], (DMAEM −26%), and the same polymer with attached silver nanoparticles were kindly provided by Dr. Nazarova O. (Institute of Macromolecular Compounds of Russian Academy of Science, Saint Petersburg, Russia). Polymers and the synthesis of silver nanoparticles connected with polymers were described in article [27]. The cationic surfactant Azo-TMAB (see Figure 1) was kindly provided by Professor Svetlana Santer (University of Potsdam, Potsdam, Germany).

### 2.2. Synthesis of Metal Nanoparticles

**Figure 2:**
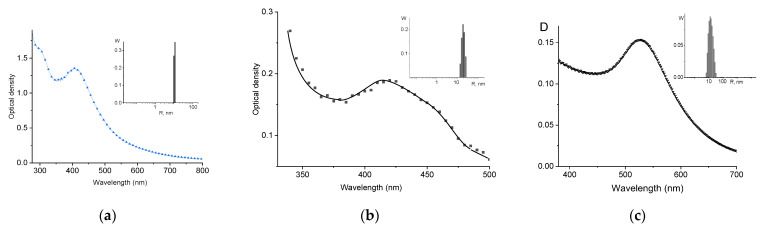
Plasmon resonance peaks and DLS data as size distributions (inserts) of palladium nanoparticles obtained by reducing a palladium ion [PdCl_4_]^2−^ in solution using a reducing agent sodium citrate (**a**) and prepared by keeping copolymer solution with K_2_PdCl_4_ (**b**) or K_2_AuCl_4_ (**c**) for about 24 h at room temperature without the addition of other chemicals. See explanation in the text. DLS data were obtained at a scattering angle of 90°.

We used the same procedures for preparing the silver and gold nanoparticles as described in articles [7,28]. The following composition of reagents was used for the synthesis of palladium nanoparticles in solution: the concentration of K_2_PdCl_4_ was [K_2_PdCl_4_] = 0.25 mM, and the concentration of reducing agent NaBH_4_ was 1.5 mM. The plasmon resonance peak and size distribution of palladium particles obtained in our research are shown in Figure 2a.

We applied the method of preparing the gold and palladium nanoparticles conjugated with polycationic polymers without the usage of reducing agents and stabilizers like it was reported in [27] for polymer–silver nanoparticle composites. The negatively charged complex ions of palladium [PdCl_4_]^2−^ or gold [AuCl_4_]^2−^ interact with polycations. In time, metal nanoparticles associated with polymer appear in the solutions. The optimal ratio of the molar concentrations of the copolymer poly[MAG–DMAEMA] and K_2_AuCl_4_ or K_2_PdCl_4_ in a solution (5:3 for gold and 4:3 for palladium) was determined by the examination of a series of solutions with the same concentration of metal salts (5 × 10^−5^ M) and different concentrations of the copolymer from 6 × 10^−7^ M to 6 × 10^−5^ M. The well-defined plasmon resonance peaks and narrow particle size distributions presented in Figure 2 indicate the formation of nanoparticles in solutions.

Methods for the synthesis of silver and gold nanoparticles on DNA were described previously [28]. For the preparation of palladium nanoparticles linked to DNA in 0.005 M NaCl, we used [DNA] = 0.005% = 1.5 × 10^−4^ M (bp) and [K_2_PdCl_4_] = 5 × 10^−5^ M. In this case, sodium citrate was used as a reducing agent at a concentration of 1.6 × 10^−4^ M.

### 2.3. Spectral Measurements

UV absorption spectra of DNA; complexes of DNA with nanoparticles, surfactants and polymers; and plasmon resonance peaks of nanoparticles were recorded using an SF-56 Spectrophotometer (Russia) and Specord 200 Plus.

### 2.4. Dynamic Light Scattering (DLS)

The Photocor Complex with DynaLS software (Russia) was used with a 654 nm laser, and a cuvette with 8 mm diameter in immersion.

### 2.5. Atomic Force Microscopy (AFM)

In the AFM method, the images of DNA and its composites were obtained on a freshly split mica surface with fixed DNA using Mg^2+^ ions. A drop of DNA solution with Mg^2+^ ions (10^−4^ M) was placed on the mica surface and kept for 10 min. After that, the surface was washed with distilled water and dried for 30 min in a vacuum dryer. The Nanoscope IV Bruker atomic force microscope was used.

### 2.6. Low-Gradient Viscometry (LGV)

The relative viscosity of DNA solutions, η_r_ = η/η_0_ (where η and η_0_ are the solution and the solvent viscosity, respectively), was measured at different velocity gradients g in the range of g = (0.5 ÷ 2) s^−1^. The contribution to the measured η_r_ value of the other components in the solution, including synthetic polymers, was negligible due to the huge molecular mass of the DNA used (M = 9 × 10^6^). The absence of gradient dependence was observed for all the systems under study. The reduced viscosity of solutions, ῃ_red_ = (η_r_ − 1)/C, was calculated; C is the concentration of DNA. A low-gradient rotational viscometer was used. The principal device is detailed in [29].

### 2.7. Flow Birefringence

The birefringence values Δn for solutions with different DNA concentrations C were measured in the field of the velocity gradient g, which provides the orientation of ellipsoidal molecular coils. The value, (Δn/g)/(η_r_ − 1)η_0_ for g → 0, was used to calculate the optical anisotropy of the statistical segment of DNA molecule (α_1_ − α_2_), i.e., the difference between the polarizabilities of the segment along (α_1_) and across (α_2_) the axis of the DNA helix. The value of (α_1_ − α_2_) gives the DNA persistence length value *p*, since (α_1_ − α_2_) = (*2p/l*)Δβ. The projection of the length of a pair of bases in the direction of the axis of the helix *l* and the optical anisotropy of a base pair Δβ along and across this direction are also included in this equation. All hydrodynamic measurements were performed at a temperature of 21 °C.

## 3. Results

We used two different methods for the fabrication of nanosized structures of DNA connected with metal nanoparticles. First, the reduction in metal ions after their binding to DNA with further DNA packaging induced by condensing agents was applied. Second, the cationic polymers with attached metal nanoparticles were added to the solutions for DNA condensation via the formation of interpolyelectrolyte complexes. The second way involves either the usage of ready-made polymers, the process of whose synthesis was described in [20], or the preparation of new conjugates. The conjugates of polymers with gold and palladium nanoparticles were prepared in our research.

For the first method, the binding of the noble metal ions to DNA was obtained by mixing the DNA solution with a solution of AgNO_3_, K_2_PdCl_4_ or K_2_AuCl_4_. Since the DNA molecule is a highly charged polyion, the ionic strength plays an important role in its conformational changes. To control this value, we introduced 0.005 M NaCl (0.005 M NaNO_3_ for solutions containing silver ions) as a supporting electrolyte into all solutions containing DNA. Indeed, the DNA sample is sodium salt, and thus, in water, the macromolecule is surrounded by its own counterions. The concentration of Na^+^ changes with the variation in DNA concentration, but taking into account the range of DNA concentrations, this change is neglected in 0.005 M NaCl. In addition, in 0.005 M NaCl we can ignore the change in the persistent length of DNA due to electrostatic interactions [30].

**Figure 3:**
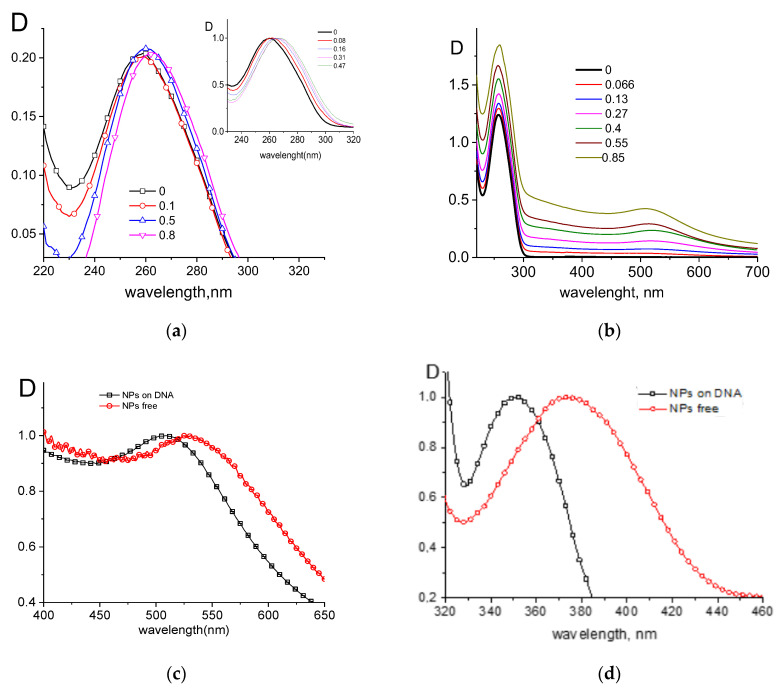
Absorption spectra of DNA and its complexes with gold ions in 0.005 M NaCl (**a**), where insert shows normalization to the peak maximum for DNA absorption spectra at different r values (r values are shown near lines); same spectra after the addition of reducing agent into DNA solution with gold ions (**b**); and comparison of the plasmon resonance peaks for nanoparticles attached to DNA, and free gold (**c**) and palladium (**d**) nanoparticles.

The binding of metal ions, including coordination compounds, with DNA bases in the major groove is always accompanied by characteristic changes in the DNA absorption spectrum. A bathochromic shift of the band and the gradual appearance of hyperchromism were observed. Figure 3a shows such spectral changes for DNA binding with gold ions in 0.005 M NaCl at different r values (r is the ratio of molar concentrations of K_2_AuCl_4_ and DNA base pairs). Similar data were obtained for DNA complexes with palladium and silver ions. After keeping the DNA solutions with noble metal ions for about 4 h at room temperature (this time is usually necessary for the coordination of ions with DNA bases) [31]), a reducing agent was added and the solution was left for another 2 h (the time required for the growth of nanoparticles on DNA [32]).

The emergence of metal nanoparticles linked to DNA was controlled by the occurrence and position of the plasmon resonance peak (see, for example, Figure 3).

**Figure 4:**
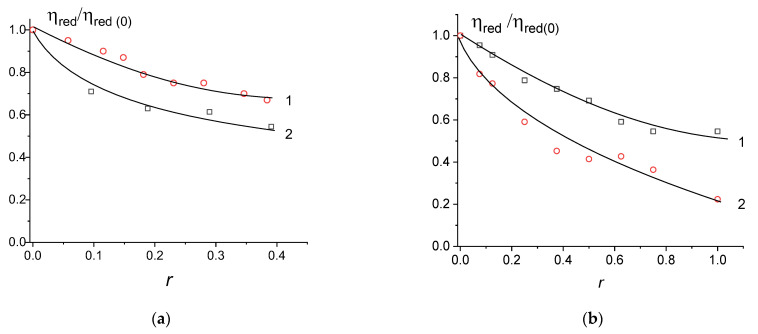
Relative changes in reduced viscosity of DNA solutions on r for DNA complexes with ions (1) and after the forming of nanoparticles connected with a macromolecule (2) in 0.005 M NaCl for gold (**a**) and silver (**b**) nanoparticles.

The volume of the DNA coil in a solution with attached nanoparticles is less for that of free DNA and DNA in complexes with appropriate metal ions, but it is still not small enough (see viscometric data presented in Figure 4). The DNA persistent length *p* does not change (we carried out the same experiments that have been described previously in [28]). Indeed, the combination of flow birefringence and viscometry give the value (Δn/g)/(η_r_ − 1)η_0_ = −(23 ± 2) × 10^−8^ cm·s^2^/g for free DNA in 0.005 M NaCl and −(21 ± 2) × 10^−8^ cm·s^2^/g and −(22 ± 2) × 10^−8^ cm·s^2^/g for DNA with gold and palladium nanoparticles, respectively. The constancy of this value indicates the invariability in the optical anisotropy of the statistical segment of DNA molecule (α_1_–α_2_) and in DNA persistence length *p* = (50 ± 5) nm for free DNA and for DNA connected with gold and silver nanoparticles in 0.005 M NaCl. The packaging of such DNA in solution can be induced by the addition of a cationic polymer or surfactant.

**Figure 5:**
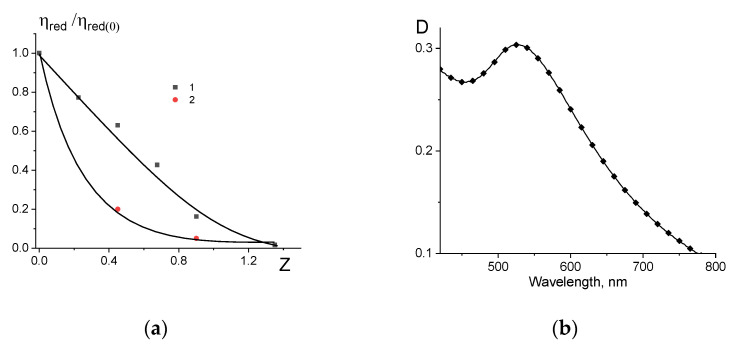
Relative change in the reduced viscosity of solutions of DNA (1) and DNA conjugates with gold nanoparticles (2) with an increase in Z value (**a**) and plasmon resonance peaks for complex (DNA + AuNPs + surfactant) system (**b**). DNA and surfactant have no absorption at examined wavelength area.

**Figure 6:**
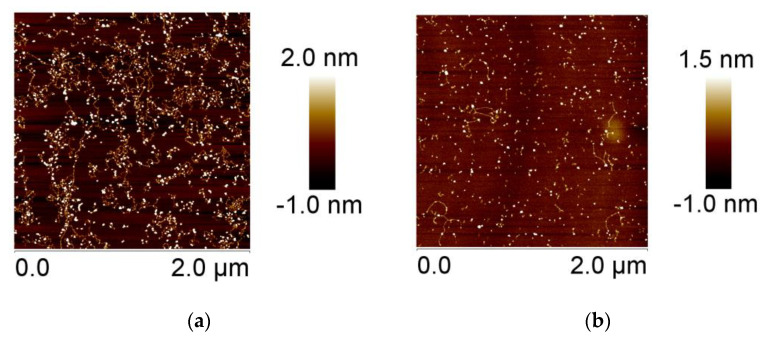
AFM images of DNA conjugates with gold nanoparticles (**a**) and compact structures after the addition of surfactants to solutions with (DNA–gold NPs) (**b**).

Previously, we studied in detail the interaction of the DNA molecule with the used cationic surfactant [33]. It was shown that in solutions of low ionic strength at a charge ratio of Z = 1 (Z is the ratio of the molar concentration of a surfactant with one positive charge to the molar concentration of DNA phosphate groups with a negative charge), the compaction of DNA in solution is observed. One can see (Figure 5a) that the same result was obtained for the free DNA and surfactant in our research. For DNA connected with gold nanoparticles, a very significant drop in viscosity was observed. In other words, the conjugation of DNA with gold nanoparticles, which leads to a decrease in the volume of the macromolecule (Figure 4a), does not prevent further DNA packaging induced by surfactants. AFM images for DNA–gold nanoparticle composites and compact structures (DNA + NPs + surfactant), as shown in Figure 6, are consistent with the viscometric data.

**Figure 7:**
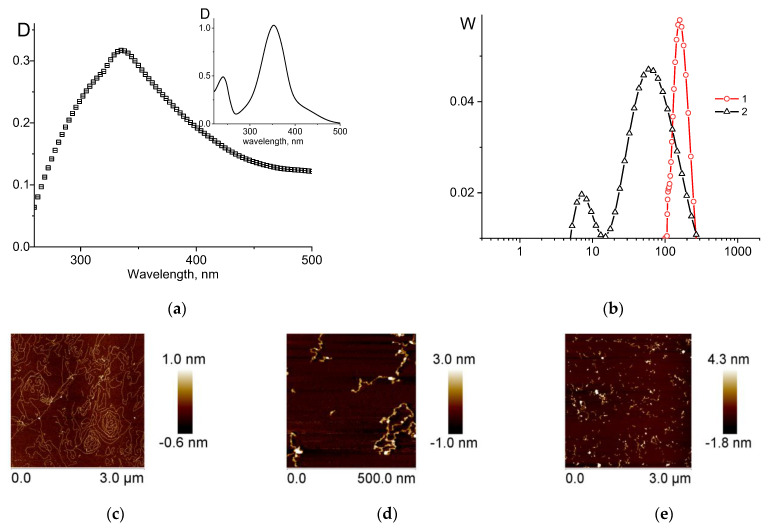
Calculated plasmon resonance peak for palladium nanoparticles in (DNA + PdNPs + surfactant) system; insert—absorption spectra of surfactant in 0.005 M NaCl (**a**) and size distribution for (DNA + PdNPs) (1) and (DNA + PdNPs + surfactant) (2) systems from DLS data (**b**). Measurements in DLS experiment were carried out at the angle 90°. AFM images of DNA on mica are also presented: free DNA (**c**); DNA conjugated with palladium nanoparticles (**d**); and (DNA + PdNPs + surfactant) system, (**e**).

Similar data were obtained for DNA conjugates with palladium nanoparticles. Note that the surfactant has an absorption peak intersecting with the plasmon resonance band (see Figure 7a), while DNA above 300 nm has no absorption. This circumstance forces us to present the calculated plasmon resonance peak for palladium nanoparticles in a complex system (DNA + surfactant + nanoparticles). Figure 7b indicates that the surfactant induces the packaging of DNA conjugates with palladium nanoparticles. The additional fast mode in regard to the size distribution can indicate the presence of free nanoparticles with a hydrodynamic radius of about 7 nm. Here, we do not present the data obtained using the DLS method for free DNA in a 0.005 NaCl solution, since in this case the intensity correlation functions at different angles and different DNA concentrations must be shown. We repeatedly studied a given DNA sample via DLS and usually obtained a bimodal dependence, where the main slow mode gives the translational diffusion coefficient of the macromolecule. The estimate of the hydrodynamic radius of the macromolecule R_H_ in this case is (450 ± 50) nm. One can see that for DNA connected with palladium nanoparticles R_H_ = (160 ± 30) nm and the DNA after packaging induced by the surfactant is R_H_ = (60 ± 20) nm. The presence of a small fraction of free nanoparticles in solution can apparently be explained by a kind of competition for contacts with nanoparticles between surfactants and DNA. AFM images of DNA conjugated with palladium nanoparticles are presented in Figure 7d,e.

**Figure 8:**
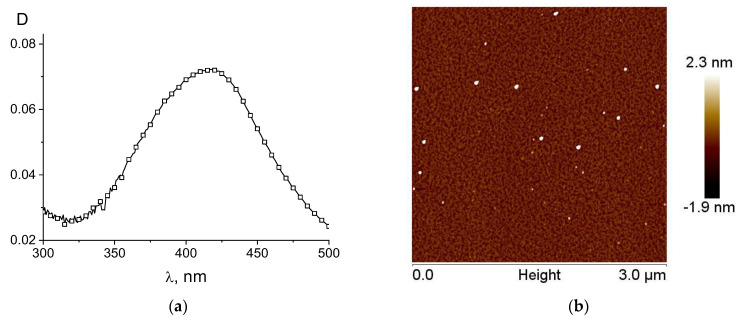
Plasmon resonance peak (**a**) and AFM image for poly-DMAEM-Ag0 on mica (**b**).

Thus, the first method for the packaging of DNA associated with noble metal nanoparticles leads to a quite satisfactory result. Let us consider the results obtained using the second method, in which DNA compaction was induced by a cationic polymer associated with nanoparticles. As noted above, for this purpose, we used ready-made cationic poly-DMAEM conjugated with silver nanoparticles (poly-DMAEM-Ag0) and poly-Mag-DMAEM copolymers conjugated with gold and palladium nanoparticles that were preliminarily prepared in our research (see the Section 2). It is known that cationic polymers induce DNA condensation in a solution. The polymer poly-DMAEM-Ag0 has a plasmon resonance peak for silver nanoparticles at 440 nm (Figure 8a). Its AFM image (Figure 8b) shows the presence of nanoparticles.

**Figure 9:**
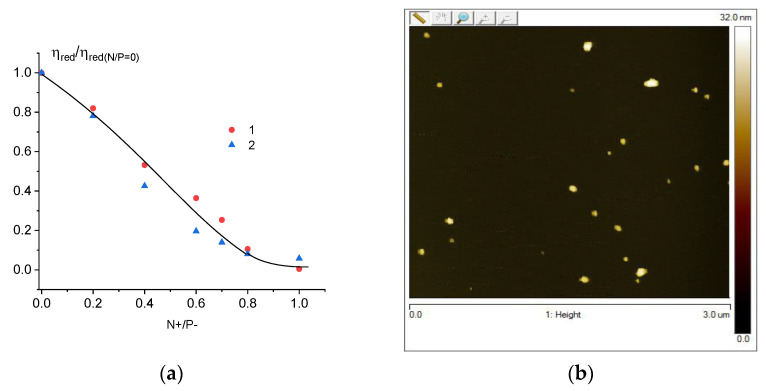
Relative change in viscosity of DNA solution with charge ratio N+/P− (**a**) for DNA interpolyelectrolyte complexes with poly-DMAEM (1) and poly-DMAEM-Ag0 (2), and AFM image of DNA complexes with poly-DMAEM-Ag0 at N+/P− = 1.3 (**b**).

Figure 9a demonstrates DNA packaging induced by poly-DMAEM- and poly-DMAEM-Ag0 in a 0.005 M NaNO_3_ solution. The type of relative change in the reduced viscosity of DNA solutions based on the charge ratio is approximately the same for DNA complexes with a polymer conjugated with nanoparticles and DNA complexes with a polymer without nanoparticles. This means that the charge properties of the polymer do not fundamentally change when conjugated with nanoparticles, and a polymer can induce DNA packaging, as one can see from the AFM data (Figure 9b). The presence of silver nanoparticles in such structures was proved earlier [34].

**Figure 10:**
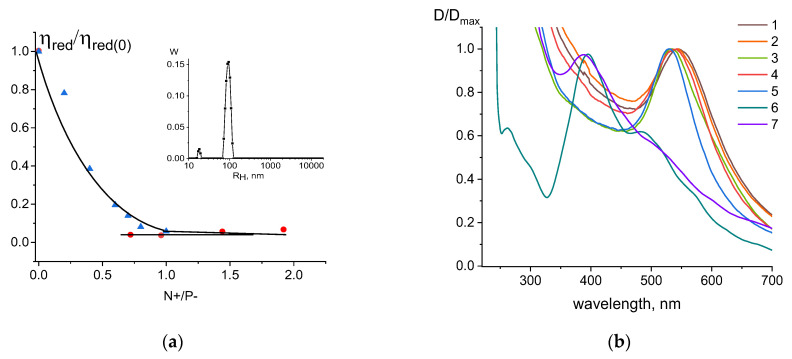
(**a**) Dependence of relative change in reduced viscosity on charge ratio N+/P− for DNA solutions with poly(MAG-DMAEM-Ag0) (1) and [DNA + AuNPs + poly(MAG-DMAEM-Ag0)] (2); insert—size distribution from DLS data for [DNA + AuNPs+ poly(MAG-DMAEM-Ag0)] system; (**b**) normalized to maximum plasmon resonance peaks for silver(1,2) and gold(3–7) nanoparticles in the systems: 1—DNA + poly(MAG-DMAEM-Ag0), N/P = 0.8; 2—poly(MAG-DMAEM-Ag0), C(DMAEM) = 1.4 × 10^−4^ M; 3—[DNA + AuNPs+ poly(MAG-DMAEM-Ag0)], N/P = 0.6; 4—[DNA + AuNPs+ poly(MAG-DMAEM-Ag0)], N/P = 1.6; 5—[AuNPs+ poly(MAG-DMAEM-Ag0)], N/P = 1.2; 6—[DNA + AuNPs]; 7—AuNPs.

Finally, we fabricated the nanosized structure on the basis of DNA–polycation complexes with silver and gold nanoparticles inside. We used the copolymer poly(MAG-DMAEM) with attached silver nanoparticles, designated as poly(MAG-DMAEM-Ag0). After the preliminary binding of DNA with gold ions (using a solution of salt K_2_AuCl_4_), we carried out the reduction in the DNA-bound ions with NaBH_4_. As a result, the solution of the DNA associated with gold nanoparticles was mixed with the solution of the polymer poly(MAG-DMAEM) that was associated with silver nanoparticles. At the charge ratio N+/P− >1, one can see DNA condensation. The size of the formed DNA nanosized structures with a polymer and two types of nanoparticles was controlled using viscometry and dynamic light scattering methods. Figure 10 shows that small particles of almost the same size were formed. The solution also contains a small fraction of free nanoparticles that do not bind to DNA. The spectral data show that the formed systems exhibited plasmon resonance peaks for both gold and silver nanoparticles. In this case, the shift in the peak for gold nanoparticles unambiguously indicates their binding to DNA.

**Figure 11:**
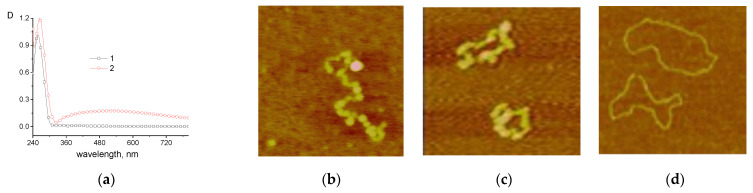
The emergence of plasmon resonance band in spectrum of DNA solution with AgNO_3_ after 7 days of storage (2); for comparison, the spectrum obtained just after preparing the solution is shown (1). [DNA] = 7.5 × 10^−5^ M, [AgNO_3_] = 10^−3^ M (**a**); AFM images of linearized (**b**) and circular (**c**,**d**) plasmid DNA pFL44/EcoRI on mica: the structures formed in DNA solution with K_2_PdCl_4_ after 4 days of storage (**b**,**c**) and free DNA (**d**). Size of images: 500 nm × 500 nm.

Figure 11 shows the not yet fully understood results in regard to the possible reduction in silver and palladium in DNA solutions without the addition of a reducing agent, where ions were bound to a macromolecule and the solutions were kept for 4–5 days. One can see the emergence of the plasmon resonance band of silver nanoparticles in the spectrum of the DNA solution with AgNO_3_ after 7 days of storage. Structures like palladium nanoparticles on DNA were observed when the palladium ions bound to DNA (solution was kept for 4 days).

The experimental results obtained in this work unambiguously show that both methods of packaging DNA that is conjugated with metal nanoparticles provide good results. Moreover, discrete small DNA particles with two types of nanoparticles (gold and silver) can be formed (Figure 10). The size of the formed structures allows us to hypothesize that they can easily penetrate the cell.

## 4. Discussion

Let us consider some issues related to the synthesis of nanoparticles on the DNA matrix. At the first stage of the fabrication of nanoparticles connected with macromolecules, the binding of the ions of noble metals to DNA was obtained. We must emphasize that after the dissociation in the water solution of K_2_PdCl_4_ or K_2_AuCl_4_, the negatively charged ions [PdCl_4_]^2−^ and [AuCl_4_]^2−^ could not interact with negatively charged DNA. Nevertheless, as a result of the aquatation, the chloride ions from the coordination sphere of ions are gradually replaced by water molecules, and positively charged complex ions [Pd(H_2_O)_4_]^2+^ and [Au(H_2_O)_4_]^2+^ can interact with DNA. At the first stage, electrostatic forces contribute to the attraction between ions and DNA, and then stronger bonds can form. Atomic groups of DNA can replace water molecules in the coordination sphere of the ion. It is important to emphasize that the binding of metal ions to DNA bases (not to oxygen atoms of phosphate groups) plays a decisive role. Indeed, the oxygen of the phosphate group cannot compete with the oxygen of a water molecule in the coordination sphere of ions. The heterocyclic nitrogen atoms of the DNA bases can form strong coordination bonds with silver, gold and palladium. Although Ag(I) is frequently thought of as having a strong preference for linear coordination, it was reported that in some cases two-coordinate linkages can be formed. The N7 atoms of purine bases in the major groove of DNA are the most suitable points for coordination. The advantage of N7-guanine creates the nearest O6 atom (guanine can act as a bidentate ligand).

Previously, different complexes were observed in DNA solutions depending on the concentration of Ag^+^ and Au^2+^ ions [28,32]. Although the electrostatic binding of metal ions to phosphate groups is observed at all stages, this binding does not play an important role in the further formation of metal nanoparticles. The excess of binding sites on DNA bases allows for the coordination of metal ions to DNA in the major groove. After filling such binding sites, the N3 cytosine starts to participate in the ion–DNA interaction as a result of local destabilization of the double helix (usually N3 cytosine is involved in the hydrogen bond between complementary bases). This exact type of binding within the double helix is preferable for the initial reduction in metal ions on DNA, with the formation of “seeds” for the further growth of nanoparticles. We can calculate the optimal concentration of metal ions in DNA solutions. Indeed, an excess of free ions from the DNA can induce the rapid growth of nanoparticles without any connection with a macromolecule. The slow growth of nanoparticles connected with DNA (more than 2 h for silver and gold nanoparticles) requires a certain deficit of free metal ions in solutions. Metal clusters and nanoparticles begin to form upon the addition of the reducing agent NaBH_4_.

DNA is a highly charged molecule, which cannot exist in a solution without other ions, usually Na^+^ ions. Therefore, to maintain the constancy of the concentration of counterions, 0.005 M NaCl was introduced into the DNA solution. This also ensures the invariance of the electrostatic component of the persistence length of DNA.

DNA condensation in an aqueous solution is a necessary step in the construction of various structures (gene vectors and different nanoplatforms) that can penetrate into the cell. As noted above, this phenomenon has been studied quite well. The problem in our study was the selection of adequate condensing agents. We settled on the use of a cationic surfactant (its role in the formation of compact DNA structures is similar to the role of lipids in the fabrication of non-viral gene vectors). When binding to DNA, surfactants have a mild effect on its structure. Indeed, electrostatic binding of the cationic “heads” to the phosphate groups of DNA occurs. The strength of this interaction is enhanced by the formation of a hydrophobic environment around the DNA double helix with the oriented uncharged “tails” of surfactants. Such binding is cooperative and does not require the penetration of the surfactant into the major groove of DNA where, as we have shown, gold, silver and palladium ions are localized. This localization is responsible for the further growth of metal nanoparticles. It is interesting to note that the growth of metal nanoparticles on a DNA matrix does not lead to the significant destabilization of its double helix. First of all, the persistence length of the macromolecule does not change, and the melting temperature of DNA only slightly decreases (we did not present these results due to the large amount of experimental data). This makes it possible to further “saturate” such compact structures with biologically active substances, for example, through the intercalation of the corresponding ligands. Here, we did not dwell on the very important enhancement of optical effects near nanoparticles, since it was considered previously [28,34].

Another condensing agent in our study was a polycationic polymer conjugated with silver nanoparticles. In this case, the formation of the interpolyelectrolyte complexes automatically involved metal nanoparticles inside the formed compact structures. We used two polymers combined with silver nanoparticles: poly-DMAEM-Ag0 and its copolymer poly(MAG-DMAEM)-Ag0. The advantages and disadvantages of these polymers are discussed in [20]. For gold and palladium nanoparticles, we prepared such polymers with nanoparticles ourselves, based on the described technique. These tasks were successfully solved. DNA packaging induced by such polymers in solutions with the emergence of compact nanosized structures was observed. We can use different polymers for this procedure, including the least toxic samples.

It is interesting to note that DNA itself, in principle, can act as a polymer that reduces metal ions. In particular, a preliminary experiment showed that when DNA solutions with AgNO_3_ salt are kept without the addition of other chemicals for 5 days, a weak plasmon resonance band appears in the solution (see Figure 11a). In addition, we observed the formation of structures resembling the appearance of palladium nanoparticles on DNA when studying the interaction of DNA with palladium compounds [35]. It is possible that the biological role of silver and palladium compounds is, to some extent, associated with the in vivo formation of metal nanoparticles that can perform catalytic functions. These effects undoubtedly require further in-depth study.

Regarding the main goal of the study—the development of the compaction of DNA coupled with nanoparticles—the following can be said: Wherever nanoparticles are fixed before the compaction process, whether on DNA or on a condensing agent (polymer), in both cases it is possible to obtain nanosized structures with the inclusion of metal nanoparticles. At the same time, if the task of forming multicomponent systems assumes the involvement of other biologically active agents, they can first be associated with DNA (then, it is preferable to use a polymer conjugated with nanoparticles for compaction), or one can use previously modified condensing agents.

## 5. Conclusions

In this paper, we tested the methods of DNA integration with silver, gold and palladium nanoparticles. The properties of DNA conjugates with metal nanoparticles, as determined via the methods of atomic force microscopy, spectroscopy, viscometry, dynamic light scattering and flow birefringence, were analyzed. DNA size, stability and rigidity (persistence length), as well as plasmon resonance peaks in the absorption spectra of systems were studied. The different methods for DNA condensation with metal nanoparticles were compared. The methods for the synthesis of palladium nanoparticles were tested and a new method for creating palladium nanoparticles using a synthetic copolymer without a reducing agent was proposed. The size and properties of the formed particles were compared with those of gold nanoparticles synthesized in a similar way. The possible usage of surfactants for the condensation of DNA conjugated with metal nanoparticles was confirmed. The sizes and shapes of formed DNA nanostructures with gold, palladium and silver nanoparticles were analyzed via the DLS and AFM methods. The fundamental possibility of simultaneously forming compact particles of DNA with silver and gold nanoparticles was shown.

## Data Availability

Protocols of experimental studies conducted at St. Petersburg University are kept in the Laboratory of Molecular Biophysics and can be provided if necessary.
